# A Clinical Course of Repeated Supratherapeutic Ingestion of Acetaminophen

**DOI:** 10.7759/cureus.59883

**Published:** 2024-05-08

**Authors:** Neelay Shah, Hunter Campbell, Vishal Patel, Jill Moormeier

**Affiliations:** 1 Neurology, University of Missouri Kansas City School of Medicine, Kansas City, USA; 2 Internal Medicine, University of Missouri Kansas City School of Medicine, Kansas City, USA

**Keywords:** acetaminophen toxicity, acute liver failure, chronic acetaminophen ingestion, drug-induced liver injury, glutathione depletion, hepatic encephalopathy, hepatocellular damage, liver transplantation, n-acetylcysteine, supratherapeutic ingestion

## Abstract

Acute liver failure (ALF) exemplifies a rapid decline in liver function among individuals with previously healthy livers, often manifesting through symptoms such as jaundice, confusion, and potentially life-threatening complications. Timely medical intervention, and, in severe instances, liver transplantation, are essential for enhancing outcomes and averting further deterioration. While the causes of ALF are multifaceted, in developed nations, it predominantly arises from drug-induced liver injury. Treatment primarily revolves around supportive measures, with severe cases necessitating liver transplantation. In instances where acute overdose with acetaminophen serves as the instigating factor, N-acetylcysteine (NAC) emerges as a pivotal component of management, as indicated by the Rumack-Matthew nomogram. The Rumack-Matthew nomogram guides treatment for acetaminophen overdose by correlating serum levels with the risk of liver damage. If levels exceed a set threshold, NAC is administered to prevent toxicity by replenishing glutathione. The decision to administer NAC is typically guided by this clinical tool, which aids healthcare providers in determining the appropriate course of action. NAC assumes a critical role in ameliorating the detrimental effects of acetaminophen overdose, particularly in averting liver damage, thus holding significant importance in patient care and recovery. While chronic acetaminophen overdose cases leading to ALF may also benefit from NAC, the supporting evidence remains weak. In this context, we present a case of ALF stemming from chronic acetaminophen ingestion, managed with NAC when liver transplantation was not a viable option.

## Introduction

Acute liver failure (ALF) results from an acute injury to the liver, severe enough to impair synthetic function and alter mental status without preexisting liver disease with the therapeutic dose being a maximum of 4,000 mg every 24 hours [1]. There is an extensive list of factors that can cause and/or contribute to liver failure; however, the most common cause in the United States is acetaminophen toxicity [2]. Most occurrences of ALF are from single overdoses, but ALF from chronic acetaminophen ingestion is also associated with morbidity and mortality [2]. Repeated supratherapeutic ingestion (RSTI) diagnosis can be difficult, as signs and symptoms are often nonspecific and point towards other diagnoses [3]. The therapeutic dose maximum is 4,000 mg every 24 hours [1].

Patients who take a single dose of 250 mg/kg or greater than 4 g over a 24-hour period without medical supervision are likely to acquire toxicity [4]. Severe liver toxicity is almost inevitable when a patient ingests an excess of 350 mg/kg per dose. Severe liver toxicity is defined as peak aspartate aminotransferase (AST) or alanine aminotransferase (ALT) greater than 1,000 international units (IU)/L [5]. Additionally, clinical factors such as chronic alcohol use, certain other medications, other liver diseases, poor nutritional status, genetics, age, and tobacco use also contribute to the decline in liver function [6]. For instance, regarding genetic variability, polymorphisms in genes encoding cytochrome P450 enzymes, such as CYP2E1, can affect the rate at which acetaminophen is metabolized into its toxic metabolite, N-acetyl-p-benzoquinone imine (NAPQI). Certain genetic variants may lead to increased activity of CYP2E1, resulting in the excessive production of NAPQI and subsequent liver damage. Here we present a case of ALF caused by RSTIs of acetaminophen, with the patient achieving a full recovery [7].

## Case presentation

The patient was a 47-year-old African American female with a history of gastroesophageal reflux disease (GERD), gastric Roux-en-Y bypass in 2018, and anastomotic marginal ulcer who was admitted for severe abdominal pain.

On initial questioning, she reported worsening severe epigastric abdominal pain for two months with acute worsening during the past five days, which she attributed to her history of marginal ulcers. Moreover, she had non-bloody vomitus and black stools in the two days before presentation. Her pain had been initially well controlled using therapeutic doses of acetaminophen; however, in the last week, she had required additional doses of acetaminophen to control her pain. She estimated taking one to two tablets of 500 mg acetaminophen every two to three hours over the past week (dose equivalent to 6,000-12,000 mg total/day). Upon presentation, she was mildly tachycardic but with other vital signs within normal limits. Her labs were significant for an AST of 1,899 IU/L (normal range: 8-48 IU/L) and ALT of 533 IU/L (normal range: 7-56 IU/L), indicating severe liver damage. The international normalized ratio (INR) was within the normal range at 1.0 (normal range: 0.8-1.2), suggesting preserved blood clotting function. Her hemoglobin level was 11.1 gm/dL (normal range: 12.0-15.5 gm/dL), reflecting a slightly decreased level of red blood cells.

Additionally, her serum acetaminophen level returned to 29 mcg/mL, and a urine drug screen was positive for cocaine. In response to these findings, she was initiated on pantoprazole, lactated Ringer's solution at 75 mL/hour, and N-acetylcysteine (NAC), aligning with the standard management protocol for acetaminophen overdose. The elevated AST and ALT levels underscore the severity of liver involvement, emphasizing the necessity for prompt and comprehensive medical intervention.

During her admission, she developed jaundice and a severe elevation of her hepatic transaminases within 10,000 seconds. Hyperbilirubinemia and an elevated INR suggestive of ALF during hospital day one (Table 1) were concurrently noted. She was initiated on a continuous NAC infusion of 15 mg/kg/hour for seven days. Clinical evaluation was notable for normal iron studies, a negative autoimmune panel, and negative serology for viral hepatitis and herpes simplex virus. A mesenteric Doppler ultrasound was performed and did not identify portal or hepatic vein thrombosis. With supportive fluids and NAC, her liver function steadily improved with a peak in her liver function tests (LFTs) above 10,000, INR at 3.08, and total bilirubin at 22.5 on the fourth hospital day, then followed by down trending of all labs until normal lab values were achieved. She underwent a scheduled esophagogastroduodenoscopy (EGD) after improvement in hepatic function on 8/15/23, demonstrating normal esophageal mucosa.

**Table 1:** Represents the correlation between HD with regards to Hgb, Cr, total bilirubin, AST/ALT, PT, INR, and acetaminophen levels H=high; L=low AST: aspartate aminotransferase; ALT: alanine aminotransferase; HD: hospital day; Hgb: hemoglobin; PT: prothrombin time; INR: international normalized ratio

Dates	8/9/23	8/10/23	8/11/23	8/12/23	8/14/23	8/15/23	8/16/23	8/17/23	8/18/23
HD	1	2	3	4	6	7	8	9	10
Hemoglobin (Hgb) (12.0-15.5 g/dL)	L 9.7	L 8.7	L 7.5	L 6.5	L 5.4	L 7.7	L 8.8	L 7.7	L 7.7
Creatinine (0.84-1.21 mg/dL)	L 0.76	L 0.79	L 0.83	L 0.67	L 0.69	L 0.57	L 0.77	L 0.67	L 0.73
Total bilirubin (0.1-1.2 mg/dL)	0.9	H 3.5	H 13.0	H 22.5	H 20.0	H 4.3	H 4.3	H 3.4	H 2.5
AST (8 -48 U/L)	1,899	H >10,000	H >10,000	H 8,235	H 3,636	H 169	H 93	H 62	H 55
ALT (7 -56 U/L)	H 533	H 3,125	H >5,000	H 4,264	H 3,063	H 1,217	H 935	H 694	H 546
PT (11.0 -13.0 seconds)	H 28.4	H 36.0	H 22.0	H 15.0	H 14.2	H 18.0	H 15.8	H 14.5	H 13.4
INR (0.8 -1.2)	H 2.38	H 3.05	H 1.89	H 1.28	H 1.24	H 1.56	H 1.37	H 1.26	1.17
Acetaminophen levels (mcg/mL)	20	29	<10.0	<10.0	<10.0	<10.0	<10.0	<10.0	<10.0

## Discussion

When an individual consumes acetaminophen, a biotransformation and detoxification process occurs within the liver by innate CYP450 enzymes. However, the prolonged and excessive use of acetaminophen can adversely affect the liver, resulting in significant disruptions to normal functioning leading to harmful consequences. In cases of excessive acetaminophen intake, a specific liver enzyme known as CYP2E1 metabolizes approximately 20% of the drug as 80% is detoxified by sulfation and glucuronidation [4]. Glutathione, an antioxidant present in the liver, plays a vital role in neutralizing NAPQI, a toxic metabolite, by binding to it. Excessive acetaminophen consumption depletes the liver's glutathione reserves, which, in turn, leads to an increase in free radicals within the body. To ensure safety, the US Food and Drug Administration (FDA) recommends that adults should not exceed 4,000 milligrams of acetaminophen per day and should refrain from prolonged use for more than ten days [7].

The presence of metabolites and inflammation resulting from acetaminophen-induced liver damage can disrupt cellular signaling pathways involved in liver regeneration. These pathways are typically mediated by growth factors such as hepatocyte growth factor (HGF) and transforming growth factor alpha (TGF-α) [6].

Immediate hospitalization and meticulous monitoring are imperative for patients with liver failure to maintain their physiological stability [8,1]. Priority should be given to care, involving the stabilization of vital signs and the management of complications, such as cerebral edema and changes in mental status [4]. The primary therapeutic objective is to identify and address the underlying cause of ALF [3]. In cases of a drug overdose, such as acetaminophen, the administration of NAC interventions should be considered [7]. In situations where both acetaminophen and alcohol have been chronically consumed, there is an increased induction of CYP2E1 enzymes, leading to increased NAPQI depletion leading to a rapid loss of glutathione concentration [1,5].

Immediate effects of acute acetaminophen toxicity

Acetaminophen, a ubiquitous over-the-counter pain reliever, carries a substantial risk when consumed in acute single ingestion exceeding recommended limits [7]. In standard therapeutic scenarios, acetaminophen undergoes glucuronidation and sulfation, yielding benign metabolites [9]. However, in overdoses, a significant portion is metabolized by the CYP2E1, culminating in the formation of the hazardous metabolite NAPQI [8].

The toxic mechanism involves an overwhelming of the liver's detoxification capacity, resulting in hepatocellular necrosis [10]. The severity of liver damage correlates directly with the ingested dose, and an acute overdose can swiftly progress to fulminant hepatic failure [7]. Initial symptoms are often nonspecific, encompassing nausea, vomiting, and malaise; however, as toxicity advances, patients may manifest right upper quadrant abdominal pain, jaundice, and hepatic encephalopathy [11]. The Rumack-Matthew nomogram is a graphical tool used to assess the risk of liver injury in cases of acute acetaminophen overdose. The nomogram plots serum acetaminophen levels against time elapsed as ingestion. If the plotted concentration surpasses a predetermined "treatment line," NAC therapy is recommended to prevent liver damage. NAC gradually replenishes depleted glutathione stores, neutralizing NAPQI, and eventually leads to hepatic recovery. Utilizing the nomogram enables timely intervention and improves patient outcomes by reducing the risk of hepatotoxicity [11].

The primary intervention for acute acetaminophen overdose is the timely administration of NAC, crucial for replenishing glutathione levels and neutralizing the toxic metabolite [11]. The early initiation of NAC is paramount for a positive outcome, underscoring the significance of prompt medical attention in cases of overdose [9].

Subacute or chronic toxicity resulting from multiple acetaminophen doses

In contrast to acute single ingestion, subacute or chronic toxicity emerges from the cumulative effect of multiple doses of acetaminophen over time, surpassing recommended limits [8]. With repeated use, there is an inherent risk of gradual metabolite accumulation, particularly the toxic NAPQI, within the liver [10].

This form of toxicity may present challenges in early detection, as symptoms tend to be subtle and insidious [11]. Prolonged exposure to elevated acetaminophen metabolite levels contributes to ongoing hepatocellular damage, with chronic users possibly experiencing persistent, low-grade symptoms such as fatigue, malaise, and gastrointestinal discomfort [12].

The management of subacute or chronic toxicity necessitates discontinuation of acetaminophen use and vigilant monitoring of liver function [9]. Healthcare providers might consider interventions aimed at enhancing liver detoxification processes [7]. Recognizing the potential harm from prolonged acetaminophen use underscores the importance of adhering to recommended dosages and consulting healthcare professionals if persistent symptoms arise [8].

A study involving 71 hospitalized individuals who had overdosed on acetaminophen was separated into two groups based on causality: those who had accidentally consumed an excessive amount and those who had intentionally used it for self-harm [5]. Surprisingly, the group that accidentally overdosed exhibited a higher incidence of severe liver-related complications, hepatic encephalopathy, and fatalities compared to the individuals who deliberately poisoned themselves [3]. This contrast persisted although the intentionally self-poisoned group had ingested substantial quantities of acetaminophen [1].

These unexpected findings highlight the relationship between the method of acetaminophen ingestion and its subsequent impact on the liver [4]. This suggests that different pathways may lead to liver damage based on the clinical circumstances [2].

## Conclusions

Most cases of ALF seen in the United States are caused by acetaminophen toxicity, typically from single-use overdoses. If discovered in a timely manner, the use of NAC has proven to be beneficial in the recovery of such patients. Compared to its use in acute acetaminophen overdose, the use of NAC has not been well documented in supratherapeutic acetaminophen ingestions, suggesting a possible difference in the pathways leading to liver failure in each circumstance. This case report highlights acute liver failure in a patient who consumed acetaminophen in repeated supratherapeutic dosages and the use of NAC in the subsequent management of her care. Further research will need to be conducted to determine if a difference truly exists in the development of liver damage from acute versus subacute supratherapeutic acetaminophen ingestion.

## References

[1] Begriche K, Penhoat C, Bernabeu-Gentey P, Massart J, Fromenty B (2023). Acetaminophen-induced hepatotoxicity in obesity and nonalcoholic fatty liver disease: a critical review. Livers.

[2] Bernal W, Auzinger G, Dhawan A (2010). Acute liver failure. Lancet.

[3] European Association for the Study of the Liver (2011). EASL clinical practice guidelines: management of hepatitis C virus infection. J Hepatol.

[4] Kamath PS, Wiesner RH, Malinchoc M (2001). A model to predict survival in patients with end-stage liver disease. Hepatol.

[5] Khanam A, Kottilil S (2021). Acute-on-chronic liver failure: pathophysiological mechanisms and management. Front Med (Lausanne).

[6] Kim WR, Therneau TM, Wiesner RH (2000). A revised natural history model for primary sclerosing cholangitis. Mayo Clin Proc.

[7] Krenzelok EP, Royal MA (2012). Confusion: acetaminophen dosing changes based on NO evidence in adults. Drugs R D.

[8] Fantilli A, López Villa SD, Zerega A (2021). Hepatitis E virus infection in a patient with alcohol related chronic liver disease: a case report of acute-on-chronic liver failure. Virol J.

[9] Osna NA, Donohue TM Jr, Kharbanda KK (2017). Alcoholic liver disease: pathogenesis and current management. Alcohol Res.

[10] Ozturk NB, Herdan E, Saner FH, Gurakar A (2023). A comprehensive review of the diagnosis and management of acute liver failure. J Clin Med.

[11] Polson J, Lee WM (2005). AASLD position paper: the management of acute liver failure. Hepatology.

[12] Chan AC, Fan ST, Lo CM (2009). Liver transplantation for acute-on-chronic liver failure. Hepatol Int.

