# Correction: A Clinical Course of Repeated Supratherapeutic Ingestion of Acetaminophen

**DOI:** 10.7759/cureus.c197

**Published:** 2024-10-09

**Authors:** Neelay Shah, Hunter Campbell, Vishal Patel, Jill Moormeier

**Affiliations:** 1 Neurology, University of Missouri Kansas City School of Medicine, Kansas City, USA; 2 Internal Medicine, University of Missouri Kansas City School of Medicine, Kansas City, USA

This article has been corrected to remove figure 1 as the authors did not obtain a license to reproduce this image prior to publication. (Figure 1 was described in the article as a representative image and was not presented as being part of this case.)

